# All Unhappy Childhoods Are Unhappy in Their Own Way—Differential Impact of Dimensions of Adverse Childhood Experiences on Adult Mental Health and Health Behavior

**DOI:** 10.3389/fpsyt.2018.00198

**Published:** 2018-05-23

**Authors:** Anna L. Westermair, Anne M. Stoll, Wiebke Greggersen, Kai G. Kahl, Michael Hüppe, Ulrich Schweiger

**Affiliations:** ^1^Department of Psychiatry and Psychotherapy, University of Lübeck Lübeck, Germany; ^2^Department of Internal Medicine I, University of Lübeck Lübeck, Germany; ^3^Department of Psychiatry, Social Psychiatry and Psychotherapy, Hannover Medical School, Hannover, Germany; ^4^Department of Anesthesiology and Intensive Care, University of Lübeck Lübeck, Germany

**Keywords:** adverse childhood experiences, ACE questionnaire, social determinants of mental ill-health, social determinants of health behavior, child maltreatment, child neglect, sexual abuse, household dysfunction

## Abstract

Adverse childhood experiences have consistently been linked with poor mental and somatic health in adulthood. However, due to methodological restraints of the main lines of research using cumulative or selective models, little is known about the differential impact of different dimensions of adverse childhood experiences. Therefore, we gathered data from 396 psychiatric in-patients on the Adverse Childhood Experiences (ACE) questionnaire, extracted dimensions using factor analysis and compared this dimensional model of adverse childhood experiences to cumulative and selective models. *Household Dysfunction* (violence against the mother, parental divorce, substance abuse or incarceration of a household member) was associated with poor health behaviors (smoking, alcohol dependency and obesity as proxy marker for an imbalance between energy intake and physical activity) and with poorer socio-economic achievement (lower education and income) in adulthood. The previously reported associations of maltreatment and sexual abuse with these outcome criteria could not be corroborated. Both *Maltreatment* (emotional and physical neglect and abuse) and *Sexual Abuse* predicted BPD, PTSD and suicidal behavior. However, the two ACE dimensions showed sufficiently divergent validity to warrant separate consideration in future studies: *Maltreatment* was associated with affective and anxiety disorders such as social phobia, panic disorder and major depressive disorder, whereas *Sexual Abuse* was associated with dysregulation of bodily sensations such as pain intensity and hunger/satiation. Also, we found both quantitative and qualitative evidence for the superiority of the dimensional approach to exploring the consequences of adverse childhood experiences in comparison to the cumulative and selective approaches.

## Introduction

Starting with the seminal study by Felitti et al. (1), adverse childhood events have been the focus of a multitude of studies which have associated them with outcomes in adulthood as diverse as panic attacks, obesity, number of bone fractures and delinquency (1–5). The majority of these previous studies can be broadly grouped into two lines of research. In the selective approach, studies focus on one particular adverse childhood experience (e.g., sexual abuse) while not controlling for others. As adverse childhood experiences are highly intercorrelated (6, 7), the reliability of results from this line of research is limited by the missing variable problem (e.g., a significant correlation between emotional neglect and obesity might be a statistical artifact due to a high correlation of emotional neglect with another adverse childhood experience which in turn exerts a causal effect on obesity). In contrast, studies employing the cumulative approach use a composite score from psychometric instruments such as the *Adverse Childhood Experiences* (ACE) questionnaire or the *Childhood Trauma Questionnaire* (8) to quantify early traumatization on a more abstract level. While this approach has the advantage of accounting for most adverse childhood experiences and therefore has been successful in highlighting the importance of childhood adversity, it does not account for the distinctiveness of adverse childhood experiences. Therefore, the cumulative approach cannot differentiate between specific types of adverse experiences regarding the specific outcomes they influence, the strength of the associations and the underlying mechanisms. This implicitly assumes that types of adverse childhood experiences are interchangeable, all having the same impact on the same outcomes through the same mechanism. Taking into account the well-documented diversity of adverse childhood experiences regarding type, timing, duration and intensity, this assumption seems dubious. Also, regardless of approach, most studies on adverse childhood experiences assess outcomes on the symptom level using self-report questionnaires, resulting in less reliable findings than assessment on disorder level using structured clinical interviews administered by mental health professionals. These conceptual and methodological problems have led the World Health Organization to call for further “population-based surveys, to capture (…) the association between past maltreatment, high-risk behavior and current health status” (9). Accordingly, the aims of this study were (a) to quantify the differential impact of distinct dimensions of adverse childhood experiences on adult mental health and health behavior while accounting for their co-occurrence and (b) to compare the cumulative, the selective and the dimensional approach regarding their predictive power for adult mental health and health behavior.

## Study population and methods

### Sample

After approval of the study design by the ethics committee of the University of Lübeck, a full survey of inpatients in the Department of Psychiatry and Psychotherapy and the Department of Psychosomatic Medicine and Psychotherapy from August 2005 to January 2007 was conducted. Exclusion criteria were mental retardation, pregnancy (due to other measurements published elsewhere) and being under-age. 455 patients gave written informed consent, in accordance with the Declaration of Helsinki. Due to later exclusions for withdrawal of informed consent or incompleteness of data, the final sample size was 396 The psychiatric morbidity was assessed using the German version of the Structured Clinical Interview for DSM-IV Disorders (10).

### Actuarial and somatic variables

Body composition was assessed with a TANITA body fat monitor (11). Self-reported smoking habits were converted into pack years (PY) as follows:

$$\begin{array}{l} \text{PY}={\left(\text{cigarettes per day}\right)}^{*}\left(\text{years of smoking}\right)/20. \end{array}$$

Participants reported their highest achieved level of school education using the predetermined categories “no graduation,” “*Sonderschule*” (graduation from Special Education School), “*Hauptschule*” (graduation after 8 years of school), “*Realschule*” (graduation after 9 years), “*Fachhochschulreife*” (graduation after 12 years, qualification for applied university entrance), “*Hochschulreife*” (graduation after 13 years of school, general qualification for university entrance). For ease of analysis, we re-coded these data into the binary variable “level of education,” with graduations after 9 years of school or more being coded as 1 and v.v. Participants reported their current employment status using the predetermined categories “fulltime employment,” “part-time employment,” “occasional employment,” “homemaker,” “training/education,” “state subsidized employment,” “unemployed,” “invalidity pension,” and “old-age pension.” We re-coded these data into the binary variable “earning capacity” with “occasional employment,” “state subsidized employment,” “unemployed,” and “invalidity pension” being coded as 0 and the other employment categories as 1. Participants rated their gross monthly household income using the categories “below 1,000 €” (~1,180 US$), “1,000–2,500 €” (~2,950 US$), “2,500–5,000 €” (~5,900 US$) and “above 5,000 €” (~5,900 US$), which was re-coded into the binary variable “household income” with income rated above 1,000 € coded as 1 and v.v.

### Psychometric instruments

Questionnaires were completed during the inpatient treatment at a pace adapted to patient needs, typically over several days. Early traumatization was retrospectively assessed with the widely used ACE questionnaire (1). It comprises 28 items that are aggregated to 10 binary coded subscales. In addition to subscales measuring massive forms of traumatization such as *sexual abuse* (4 items) and *physical abuse* (2 items), the ACE also covers less obvious forms of traumatization such as *psychological abuse* (two Items)*, emotional neglect* (five items) and *physical neglect* (five items) as well as adverse life circumstances such as *parental separation or divorce* (one item)*, violence against the mother* (four Items)*, substance abuse of a household member* (o.a.h.m.; two items)*, mental illness o.a.h.m*. (two items) and *incarceration o.a.h.m*. (one item). The items are adapted from the Tactics Scale (12), the Wyatt Sexual History Questionnaire (13) and the National Family Violence Survey (14) and were compiled by Felitti et al. based on theoretical considerations. Commonly, the subscales are then added up to yield the ACE sum score ranging from zero to ten. In the validation study of the German version of the ACE questionnaire, a good internal consistency of 0.76 (Cronbach's α) was found as well as evidence for convergent and divergent validity (15). Concerning factorial validity, several studies found a three-factorial solution for the English version of the ACE questionnaire with the factors *Sexual Abuse, Maltreatment*, and *Household Dysfunction* (16–18). Recently, this finding has been replicated using the German version by principle component analysis with Kaiser normalization and direct oblimin rotation (Westermair et al., unpublished). According to this latent structure, we formed three ACE dimensions by adding up the items who loaded highest on the same component. The ACE subscales *parental separation or divorce, violence against the mother, substance abuse o.a.h.m*. and *incarceration o.a.h.m*. were aggregated to the dimension *Household Dysfunction*, while the subscales *physical abuse, psychological abuse, emotional neglect* and *physical neglect* formed the dimension *Maltreatment* and the subscales *sexual abuse and mental illness o.a.h.m*. were added up to yield the dimension *Sexual Abuse*.

Global distress was assessed with the *Symptom Checklist* (SCL-90-R) (19) in its German version (20). The 90 items cover the primary symptom dimensions somatization, obsessive-compulsive, interpersonal sensitivity, depression, anxiety, hostility, phobic anxiety, paranoid ideation and psychoticism on 5 point Likert scales. The grand mean yields the *Global Severity Index* (GSI).

Depressive symptoms were quantified using *Beck's Depression Inventory* (BDI) (21) and the *Quick Inventory of Depressive Symptoms* (QIDS) (22) in their respective German versions (23, 24). The BDI is a self-report instrument with 21 items on 4 point Likert scales, which are added up to yield a sum score (≤11: clinically unremarkable, 11–17: mild to moderate, ≥18: clinically relevant depressive symptoms). The QIDS assesses the nine dimensions of depressive symptomatology [depressed mood, loss of interest or pleasure, concentration/decision making, self-outlook, suicidal ideation, energy/fatigability, sleep, weight/appetite change, and psychomotor changes (25)] with 16 items on 4 point Likert scales (coded 0–3), which are added up to yield a sum score with a maximum of 27 (0–5: clinically unremarkable, 6–10: mild, 11–15: moderate, 16–20: severe, 21–27: very severe depressive symptoms). With the *Three-Factor Eating Questionnaire* (TFEQ) (26), eating behavior was assessed on the dimensions cognitive restraint of eating (21 items), disinhibition (16 items) and hunger (14 items). The TFEQ subscales have been shown to have very good internal consistency in various populations (range of Cronbach's α = [0.75; 0.87] as well as factorial and prognostic validity. Using the German version (27) of the *Brief Pain Inventory* (BPI) (28), mean and maximum pain intensity and mean impairment due to pain were quantified for the preceding 24 h on 11 point Likert scales.

### Statistical analysis

Statistical analysis was carried out using the *IBM*™ *Software Package for the Social Sciences* (SPSS)™ for Windows, version 23. Due to low prevalence and thus variance, the outcome criteria alcohol abuse, schizophrenia, bipolar disorder, somatization disorder, personality disorders (other than Borderline personality disorder) and attention deficit and hyperactivity disorder were excluded from further analysis. For the same reason, anorexia nervosa, bulimia nervosa and binge eating disorder were aggregated into a new outcome criterion entitled “eating disorder.” Unless otherwise specified, all tests were two-tailed with the type I error level set to 0.05. For discrete criterion variables, six logistic regression models were computed using logit as link function: the null model only including the control variables age and gender, model 1 including the control variables and the ACE sum score as predictors, models 2 to 4 including the control variables and one of the ACE dimensions (*Household Dysfunction, Maltreatment and Sexual Abuse*) and model 5 including the control variables and all three ACE dimension as predictors. R^2^ was calculated according to Cox and Snell/Nagelkerkes. Models 1–4 were compared to model 5 using the likelihood ratio test, based on only the kernel of the log-likelihood (thus excluding the constant). The test-statistic D was computed with the formula

$$\begin{array}{l} \text{D}=-2\text{ ln }\left[\text{Likelihoo}{\text{d}}_{\text{Model}1/2/3/4}/\text{Likelihoo}{\text{d}}_{\text{Model}5}\right]. \end{array}$$

D was compared to critical values obtained from tables of the χ^2^ distribution with degrees of freedom equal to the change in degrees of freedoms across models 1 and 5, i.e., $\chi_{\left(2\right)}^{2}$ = 5.99.

For continuous criterion variables, five blockwise multiple regression models were computed with the control variables in block 1 (=null model). In block 2, model 1 included the ACE sum score, models 2–4 included one of the ACE dimensions and model 5 included all three ACE dimension. To compare models 1–4 to model 5, the test-statistic F_comp_ was computed as follows:

$$\begin{array}{l} {\text{F}}_{\text{comp}}=\left[{\left(\text{N}-{\text{k}}_{\text{Model}5}-1\right)}^{*}\left({\text{R}}_{\text{Model}5}^{2}-\;{\text{R}}_{\text{Model}1/2/3/4}^{2}\right)\right]/\\ \left[\left({\text{k}}_{\text{Model}5}-\;{\text{k}}_{\text{Model}1/2/3/4}\right)\left(1-{\text{R}}_{\text{Model}5}^{2}\right)\right] \end{array}$$

with k denominating the number of predictors in the respective model. Critical values to compare F_comp_ to were obtained from tables of the F distribution with degrees of freedoms equal to those in the above given formula, i.e., *F*_(5, 2)_ = 19.30. As the F ratio is known to be influenced by the number of predictors in the model, we also computed the Akaike Information Criterion (AIC), a measure of fit that penalizes models for having more predictors (29), as follows:

$$\begin{array}{l} \text{AIC}={\text{n}}^{*}\text{Ln}\left(\text{SSE}/\text{n}\right)+2^{*}\left(\text{k}+1\right). \end{array}$$

To compensate for violation of assumptions regarding the sample distribution, resampling methods were applied where adequate (Bias corrected and accelerated with 1,000 samples and the confidence level set to 95%). Honoring the exploratory nature of the study, we refrained from correction for multiple comparisons in order to prevent type II error rate inflation.

## Results

The final sample consisted of 159 men (mean age 42.8 years, *SD* = 13.5) and 237 women (mean age 39.8 years, *SD* = 13.2). 77.6% of the sample reported an income below 2,500 € per month (~2,950 US$), in comparison to 38.1% of the general population in Germany (30). The lifetime psychiatric morbidity is given in Table 1. 60.1% of participants met criteria for more than one mental disorder (though not necessarily at the same point in their lives), with a maximum of nine disorders per participant. The most common lifetime diagnosis was Major Depressive Disorder with 58.3% of the sample meeting criteria. 33.8% of participants reported a previous suicide attempt. Descriptive statistics on the data from the psychometric instruments are presented in Table 2, descriptive statistics on the data from somatic variables are given in Table 3.

**Table 1:** Lifetime prevalence rates of mental disorders in the study sample (*N* = 396).

	**[%]**
Mild cognitive impairment	0.5
Schizophrenia	8.1
Alcohol abuse	7.3
Alcohol dependency	19.4
Bipolar disorder	3.8
Major depressive disorder	58.3
Dysthymia	18.9
Social phobia	12.1
Specific phobia	10.4
Panic disorder	11.1
Obsessive-compulsive disorder	13.4
Posttraumatic stress disorder	12.6
Anorexia nervosa	2.8
Bulimia nervosa	8.1
Binge-eating-disorder	1.6
Somatization disorder	4.5
Personality disorder	28.8
Of which: cluster A	1.0
Cluster B	23.5
Cluster C	4.3
Attention deficit and hyperactivity disorder	5.1

**Table 2:** Questionnaire data.

**Questionnaire**	**Subscale/Score**	**Mean**	***SD***
Symptom checklist revised 90 (SCL-R-90)	Global severity index (GSI)	1.5	0.8
Beck's depression inventory (BDI)	Sum score	24.9	12.7
Quick inventory of depressive symptoms (QIDS)	Sum score	14.6	5.8
Brief pain inventory (BPI)	Impairment due to pain	3.6	2.5
	Maximum pain	5.4	2.4
	Mean pain	3.9	2.0
Three-Factor Eating Questionnaire (TFEQ)	Disinhibition of eating	5.7	4.2
	Cognitive restraint of eating	7.7	5.4
	Hunger	4.5	3.7

**Table 3:** Somatic variables.

	**Unit**	**Mean**	***SD***
Body mass index (BMI)	kg/m^2^	27.2	6.1
Abdominal circumference	cm	94.7	16.1
Body fat mass	kg	24.6	14.5
Fat-free body mass	kg	55.7	11.4
Pack years (PY)	Packs/day*years	13.9	15.9

In the regression models containing all three ACE dimensions as predictors, no dimension predicted all criteria and no criterion was predicted by all dimensions. ΔR^2^ and regression coefficients are given in Table 4 for all criteria.

**Table 4:** ACE sum score and ACE dimensions, respectively, as predictors in logistic and multiple regression models.

**Criteria**		**Predictors**											
		**Model 1 (cumulative)**		**Model 2 (selective)**		**Model 3 (selective)**		**Model 4 (selective)**		**Model 5 (dimensional)**			
	**Type of regression**		**Sum score**		**Household dysfunction**		**Maltreatment**		**Sexual abuse**		**Household dysfunction**	**Maltreatment**	**Sexual abuse**
		Δ**R**^2^	***b/*****ß**	Δ**R**^2^	***b/*****ß**	Δ**R**^2^	***b/*****ß**	Δ**R**^2^	***b/*****ß**	Δ**R**^2^	***b/*****ß**	***b/*****ß**	***b/*****ß**
BMI	M	0.08^*^	0.21^*^	0.03^*^	0.19^*^	0.02^*^	0.15^*^	0.02^*^	0.13^*^	0.07^*^	0.15^*^	0.07	0.04
Abdominal circumference	M	0.03^*^	0.18^*^	0.03^*^	0.17^*^	0.01^*^	0.12^*^	0.01^*^	0.10^*^	0.03^*^	0.15^*^	0.04	0.02
Body fat mass	M	0.03^*^	0.17^*^	0.03^*^	0.17^*^	0.01^*^	0.11^*^	0.01^*^	0.11^*^	0.03^*^	0.15^*^	0.03	0.04
Fat-Free body mass	M	0.01^*^	0.09^*^	0.01^*^	0.07^*^	0.01^*^	0.08^*^	0.00	0.04	0.01	0.05	0.06	0.00
PY	M	0.01^*^	0.11^*^	0.02^*^	0.12^*^	0.01^*^	0.11^*^	0.00	0.00	0.02^*^	0.11^*^	0.08	−0.06
Alcohol Dependency	N	0.00/0.00	0.06	0.02/0.02^*^	0.29^*^	0.00/0.00	−0.02	0.00/0.00	0.08	0.02/0.03^*^	0.37^*^	−0.14	−0.00
level of education	N	0.05/0.08^*^	−0.21^*^	0.07 /0.10^*^	−0.53^*^	0.03/0.05^*^	−0.29^*^	0.01/0.01	−0.23	0.08/0.12^*^	−0.49^*^	−0.15	0.12
earning capacity	N	0.06/0.08^*^	−0.22^*^	0.05/0.07^*^	−0.47^*^	0.04/0.05^*^	−0.29^*^	0.02 /0.03^*^	−0.47^*^	0.06/0.06^*^	−0.33^*^	−0.14	−0.19
household income	N	0.03/0.04^*^	−0.15^*^	0.03/0.04^*^	−0.31^*^	0.02/0.02^*^	−0.20^*^	0.01/0.01	−0.30	0.03/0.05^*^	−0.27^*^	−0.09	−0.10
GSI	M	0.12^*^	0.36^*^	0.07^*^	0.27^*^	0.11^*^	0.33^*^	0.04^*^	0.21^*^	0.12^*^	0.12^*^	0.24^*^	0.09
Impairment due to pain	M	0.06^*^	0.25^*^	0.02^*^	0.16^*^	0.06^*^	0.24^*^	0.02^*^	0.16^*^	0.06^*^	0.05	0.18^*^	0.08
Social Phobia	N	0.02/0.04^*^	0.20^*^	0.00/0.01	0.23	0.02 /0.03^*^	0.42^*^	0.01/0.02^*^	0.45^*^	0.03/0.05^*^	−0.05	0.38^*^	0.28
Panic Disorder	N	0.01/0.02	0.12^*^	0.00/0.01	0.17	0.01/0.02^*^	0.27^*^	0.00/0.00	−0.54	0.02/0.04	0.15	0.29^*^	−0.42
Eating Disorder	N	0.02/0.04^*^	0.20^*^	0.00/0.01	0.16	0.02/0.05^*^	0.43^*^	0.02 /0.03^*^	0.61^*^	0.03/0.05^*^	−0.09	0.36^*^	0.42
MDD	N	0.04/0.05^*^	0.17^*^	0.01/0.01	0.13	0.05/0.07^*^	0.35^*^	0.01/0.02^*^	0.35^*^	0.06/0.07^*^	−0.08	0.35^*^	0.15
BDI	M	0.11^*^	0.35^*^	0.05^*^	0.22^*^	0.11^*^	0.33^*^	0.05^*^	0.24^*^	0.12^*^	0.05	0.25^*^	0.14^*^
QIDS	M	0.12^*^	0.36^*^	0.05^*^	0.22^*^	0.12^*^	0.34^*^	0.04^*^	0.22^*^	0.13^*^	0.06	0.28^*^	0.11^*^
Suicide attempt	N	0.06/0.08^*^	0.22^*^	0.02/0.03^*^	0.30^*^	0.05/0.07^*^	0.35^*^	0.04/0.05^*^	0.15^*^	0.07/0.09^*^	0.08	0.24^*^	0.40^*^
BPD	N	0.08/0.12^*^	0.40^*^	0.02/0.03^*^	0.40^*^	0.07/0.10^*^	0.69^*^	0.06/0.09^*^	1.14^*^	0.09/0.13^*^	0.06	0.52^*^	1.20^*^
PTSD	N	0.09/0.16^*^	0.42^*^	0.03 /0.06^*^	0.49^*^	0.07 /0.14^*^	0.80^*^	0.07/0.12^*^	1.21^*^	0.09/0.14^*^	0.03	0.52^*^	0.86^*^
Dysthymia	N	0.01/0.01	0.08	0.00/0.01	−0.13	0.01/0.02^*^	0.20^*^	0.01/0.02^*^	0.45^*^	0.03/0.05^*^	−0.36^*^	0.20	0.54^*^
Maximum pain	M	0.04^*^	0.21^*^	0.01	0.09	0.03^*^	0.18^*^	0.04^*^	0.21^*^	0.04^*^	−0.01	0.11	0.16^*^
Mean pain	M	0.06^*^	0.26^*^	0.02^*^	0.16^*^	0.05^*^	0.22^*^	0.05^*^	0.24^*^	0.07^*^	0.06	0.12	0.19^*^
disinhibition o.e.	M	0.02^*^	0.13^*^	0.01	0.08	0.01	0.07	0.03^*^	0.17^*^	0.02^*^	0.03	0.01	0.15^*^
cognitive restraint o.e.	M	0.01^*^	0.12^*^	0.01	0.07	0.01^*^	0.11^*^	0.01	0.07	0.02	0.01	0.10	0.03
OCD	N	0.01/0.02^*^	−0.13^*^	0.01/0.02^*^	−0.30^*^	0.01/0.01	−0.15	0.01/0.01	−0.35	0.01/0.02	−0.23	−0.03	−0.21
Specific phobia	N	0.00/0.00	0.05	0.00/0.00	0.11	0.00/0.01	0.15	0.00/0.01	−0.24	0.01/0.03	0.12	0.21	−0.47^*^
hunger	M	0.00	0.04	0.00	0.03	0.00	−0.01	0.01	0.08	0.01	0.02	−0.05	0.08

*Model 1, regression model including the control variables age and gender and the ACE sum score. Model 2–4, regression models including the control variables and one ACE dimension. Model 5, regression model including the control variables and all three ACE dimension. ΔR^2^, change in the estimate of adjusted R^2^ between the null model (only control variables as predictors) and models 1–5, respectively. In logistic regression, R^2^ was estimated according to Cox and Snell/Nagelkerkes. B, unstandardized regression coefficient; ß, standardized regression coefficient*.

* *Significant at 0.05 α error level*.

*Significant regression coefficients are shown on a gray background, the lighter gray indicating that the association did not reach significance any more upon statistical control for the other ACE dimensions. Sum score = sum score of the Adverse Childhood Experiences questionnaire (ACE). BMI, body mass index [kg/m^2^]; PY, pack years = lifetime nicotine exposure; GSI, Global Severity Index of the Symptom Checklist Revised 90 (SCL-R-90). Cognitive restraint of eating (o.e.), disinhibition of eating and hunger denominate scales of the Three-Factor Eating Questionnaire (TFEQ), maximum pain, mean pain and impairment due to pain denominate scales of the Brief Pain Inventory (BPI). Eating Disorder, Anorexia nervosa, Bulimia nervosa and/or Binge Eating Disorder. MDD, Major Depressive Disorder; BDI, Beck's Depression Inventory; QIDS, Quick Inventory of Depressive Symptoms; BPD, Borderline Personality Disorder; PTSD, Posttraumatic Stress Disorder; OCD, Obsessive-Compulsive Disorder; M, multiple linear regression; N, nominal logistic regression*.

Regarding model comparisons, discrete variables were significantly better predicted by a model containing the three ACE dimensions as predictors (model 5) than by a model containing the ACE sum score as predictor (model 1; D ε [10.16; 24.08], see Table 5), except for obsessive-compulsive disorder (OCD, D = 2.29, Δ AIC = −1.71). With continuous variables, equivalent comparisons did not reach statistical significance, but comparisons of the AIC also favored model 5 (Δ AIC ε [9.41; 49.20]) except for GSI, which was equally well predicted by both models (F_comp_ = −0.24, Δ AIC = −0.76). Also, the majority of discrete variables were significantly better predicted by a model containing all ACE dimensions as predictors than by a model containing only the ACE dimension *Household Dysfunction* as predictor (model 2 vs. 5, D ε [6.33; 43.81], see Table 5). Alcohol dependency, earning capacity, household income, obsessive-compulsive disorder and specific phobia were equally well predicted by both models. Model 5 also predicted most discrete criteria significantly better than a model with only the ACE dimension *Maltreatment* as predictor (model 3, D ε [8.91; 28.87]), but not social phobia and obsessive-compulsive disorder, which were equally well predicted by both models. Also, model 5 predicted most discrete criteria significantly better than a model with only the ACE dimension *Sexual Abuse* as predictor (model 4, D ε [9.33; 39.85]), with the exception of obsessive-compulsive disorder, which was equally well predicted by both models. Regarding continuous criteria, equivalent comparisons did not reach statistical significance, but comparisons of the AIC also favored model 5 over models 2–4 (Δ AIC ε [3.64; 68.37]).

**Table 5:** Comparison of the cumulative and selective approach with the dimensional approach.

		**Models 1 and 5**			**Models 2 and 5**			**Models 3 and 5**			**Models 4 and 5**		
**Criteria:**	**Type of regression**	**D**	**F_comp_**	**Δ AIC**	**D**	**F_comp_**	**Δ AIC**	**D**	**F_comp_**	**Δ AIC**	**D**	**F_comp_**	**Δ AIC**
BMI	M		−0.62	20.54		1.27	3.64		3.39	26.18		4.88	29.00
Abdominal circumference	M		0.00	36.48		0.47	5.75		3.78	39.15		4.96	41.60
Body fat mass	M		−0.46	34.34		0.47	5.23		3.31	37.69		3.54	37.91
Fat-Free body mass	M		0.45	27.63		1.19	4.44		1.78	26.23		4.47	29.90
PY	M		3.76	49.20		1.81	10.08		3.84	43.95		5.87	47.77
Alcohol dependency	N	17.41^*^		13.41	−5.00		−1.00	18.23^*^		14.23	18.18^*^		14.18
Level of education	N	20.99^*^		16.99	6.33^*^		2.33	29.87^*^		25.87	39.85^*^		35.85
Earning capacity	N	7.64^*^		3.64	−3.37		0.63	14.98^*^		10.98	18.97^*^		14.97
Household income	N	10.69^*^		6.68	0.15		−3.85	14.95^*^		10.95	17.07^*^		13.07
GSI	M		−0.24	−0.76		12.69	24.20		4.15	3.66		20.50^*^	39.95
Impairment due to pain	M		0.43	9.41		7.45	5.39		0.44	9.29		7.67	13.68
Social phobia	N	11.16^*^		7.16	13.92^*^		9.92	5.71		1.71	16.40^*^		12.40
Panic disorder	N	15.89^*^		11.89	6.52^*^		2.52	14.04^*^		10.04	18.99^*^		14.99
Eating disorder	N	11.21^*^		7.21	12.01^*^		8.01	9.21^*^		5.21	12.45^*^		8.45
MDD	N	17.27^*^		13.27	22.32^*^		18.32	10.63^*^		6.63	27.28^*^		23.28
BDI	M		1.21	47.40		11.34	44.24		3.38	44.80		15.93	68.37
QIDS	M		0.69	27.70		17.58	40.28		1.90	24.62		18.76	57.22
Suicide attempt	N	14.83^*^		10.83	18.73^*^		14.73	17.14^*^		13.14	22.62^*^		18.62
BPD	N	14.81^*^		10.81	43.81^*^		37.81	20.75^*^		16.75	22.13^*^		18.13
PTSD	N	19.28^*^		15.28	32.06^*^		28.06	25.29^*^		21.29	26.74^*^		22.74
Dysthymia	N	24.08^*^		20.08	19.50^*^		15.50	18.93^*^		14.93	17.49^*^		13.49
Maximum pain	M		0.41	9.70		6.85	5.78		1.87	11.22		0.41	6.23
Mean pain	M		0.00	15.24		9.78	5.76		3.91	18.95		2.61	17.18
disinhibition o.e.	M		1.90	22.98		3.02	8.34		4.32	25.56		−0.43	16.43
cognitive restraint o.e.	M		0.43	21.41		1.99	8.69		1.11	16.31		2.22	22.33
OCD	N	2.29		−1.71	1.32		−2.68	5.11		1.11	4.05		0.05
Specific phobia	N	10.19^*^		6.19	5.15		1.15	8.91^*^		4.91	9.33^*^		5.33
Hunger	M		1.05	22.13		1.63	5.99		1.83	18.27		0.61	16.94
Mean				17.32			11.05			17.99			23.57

*Model 1, regression model including the control variables age and gender and the ACE sum score (cumulative approach). Model 2–4, regression models including the control variables and one ACE dimension (selective approach). Model 5 = regression model including the control variables and all three ACE dimension (dimensional approach). D = test statistic for the likelihood ratio test = comparison between regression models for discrete criterion variables (positive values favoring model 5). F_comp_, test statistic for the comparison between regression models for continuous variables (positive values favoring model 5). Δ AIC, difference in Aikake's Information Criterion between models 1 and 5 (positive values favoring Model 5)*.

* *Significant at .05 α error level*.

*Significant regression coefficients are shown on a gray background, the lighter gray indicating that the association did not reach significance any more upon statistical control for the other ACE dimensions. Sum score = sum score of the Adverse Childhood Experiences questionnaire (ACE). BMI, body mass index [kg/m^2^]; PY, pack years = lifetime nicotine exposure; GSI, Global Severity Index of the Symptom Checklist Revised 90 (SCL-R-90). Cognitive restraint of eating (o.e.), disinhibition of eating and hunger denominate scales of the Three-Factor Eating Questionnaire (TFEQ). Maximum pain, mean pain and impairment due to pain denominate scales of the Brief Pain Inventory (BPI). Eating Disorder = Anorexia nervosa, Bulimia nervosa and/or Binge Eating Disorder. MDD, Major Depressive Disorder; BDI, Beck's Depression Inventory; QIDS, Quick Inventory of Depressive Symptoms; BPD, Borderline Personality Disorder; PTSD, Posttraumatic Stress Disorder; OCD, Obsessive-Compulsive Disorder; M, multiple linear regression; N, nominal logistic regression*.

## Discussion

As expected, the sum score of the Adverse Childhood Experiences questionnaire (ACE) proved to be a predictor of a wide array of mental and somatic risk factors, symptoms and disorders, as did the ACE dimensions when analyzed separately. However, combining the three ACE dimensions *Household Dysfunction, Maltreatment*, and *Sexual Abuse* as predictors provided us with more differentiated and also mostly statistically superior models.

### Household dysfunction

In our data, the criteria centering on obesity, i.e., BMI, abdominal circumference and body fat mass, were predicted by the ACE sum score as well as by the ACE dimensions when analyzed separately. However, after inclusion of all three ACE dimensions in one model, the previously reported and here replicated associations of *Maltreatment* or *Sexual Abuse* and obesity (31) no longer were significant. Similarly, lifetime nicotine consumption, level of education, earning capacity and household income were predicted by *Household Dysfunction* and *Maltreatment* and/or *Sexual Abuse* when analyzed separately. But when analyzed jointly, only the ACE dimension *Household Dysfunction* remained a significant predictor. Also, alcohol dependency was predicted by *Household Dysfunction*, but not *Maltreatment* or *Sexual Abuse*.

Our findings corroborate previous research on the association of adverse childhood experiences that form the *Household Dysfunction* dimension in our study with adult obesity, nicotine consumption, level of education, earning capacity, household income and alcohol dependency (32–36). However, our negative finding regarding the association of *Maltreatment* and/or *Sexual Abuse* with these criteria is in conflict with research concluding that childhood verbal, physical and sexual abuse is associated with obesity and smoking in adulthood (34, 35, 37, 38), that neglect is the strongest negative predictor for academic success (39) and that maltreatment and sexual abuse are associated with adult alcohol dependency (33, 40, 41). As neither of these studies did control for the types of adverse childhood experiences that form the *Household Dysfunction* dimension in our study, we propose that their results might be false positive due to missing variable bias. There is empirical support for this hypothesis regarding adult alcohol consumption: Widom et al. (42) found the effect of neglect, maltreatment and sexual abuse on adult alcohol consumption to be no longer significant after controlling for parental alcohol consumption (which is an ACE subscale in the *Household Dysfunction* dimension). Also, a recent meta-analysis on childhood neglect and abuse found no consistent increase in problematic alcohol use (31) and a latent class analysis found no significant increase in alcohol abuse in adult depressed outpatients who reported severe abuse and neglect in childhood (43).

To sum it up, childhood *Household Dysfunction* was associated with poor health behavior (smoking, alcohol dependency and obesity as proxy marker for an imbalance between energy intake and physical activity) and poor socio-economic achievement (lower education, earning capacity and income) in adulthood. This association might be attributable to model-based learning of dysfunctional coping strategies: Children witnessing parental substance consumption, violence, divorce, delinquency and/or incarceration lack models for constructive problem solving and functional emotion regulation. They thus are likely to become adults failing to resolve their interpersonal, academic and professional problems and consuming nicotine, alcohol or high energy food to relieve the consecutive stress. This hypothesis is supported by data from Strine et al. (44) who found the association of adverse childhood experiences with self-reported alcohol problems to be mediated by psychological distress.

### Maltreatment and sexual abuse

Impairment due to pain was predicted by the ACE sum score as well as by the ACE dimensions when analyzed separately. However, after inclusion of all three ACE dimensions in one model, only *Maltreatment* remained a significant predictor. Similarly, social phobia, panic disorder and MDD were predicted by *Maltreatment* and *Sexual Abuse* when analyzed separately. But when analyzed jointly, only *Maltreatment* remained a significant predictor. Also, eating disorders were predicted by the ACE sum score and the dimension *Maltreatment*, but not *Household Dysfunction* or *Sexual Abuse*. Severity of depressive symptoms (i.e., BDI and QIDS scores), previous suicide attempts, BPS and PTSD were predicted by the ACE sum score and the three ACE dimensions when analyzed separately. However, after inclusion of all three ACE dimensions in one model, only *Maltreatment* and *Sexual Abuse* remained significant predictors. Dysthymia and pain intensity were predicted by the ACE sum score and the ACE dimensions *Sexual Abuse, Household Dysfunction*, and/or *Maltreatment* when analyzed separately. But when analyzed jointly, only *Sexual Abuse* remained a significant predictor. Also, disinhibition of eating was predicted by the ACE sum score and the dimension *Sexual Abuse*, but not *Household Dysfunction* or *Maltreatment*.

These findings corroborate previous research on the association of childhood maltreatment with impairment due to pain, social phobia, eating disorders (31, 45–48) and sexual abuse with pain intensity (49, 50). The previously documented association between sexual abuse and eating disorders (51) showed a pronounced trend in our study but did not reach significance, possibly due to low prevalences of both sexual abuse and eating disorders in our sample. Regarding affective disorders, an association with childhood maltreatment and sexual abuse is well documented on the level of self-report depressive symptoms (52–58). As our analysis was rather based on distinct DSM-IV disorders, it yielded a more differentiated picture: Whereas *Maltreatment* predicted major depressive disorder, *Sexual Abuse* predicted Dysthymia.

To sum it up, both the ACE dimensions *Maltreatment* and *Sexual Abuse* predicted PTSD, BPD and suicidal behavior, replicating ample previous research (40, 53, 55, 57, 59–64). However, the two ACE dimensions showed sufficiently divergent validity to warrant separate consideration: *Maltreatment* was the sole predictor of social phobia, panic disorder and major depressive disorder. This association of childhood maltreatment with affective and anxiety disorders has been proposed to be mediated by deficits in emotion regulation such as anxiety sensitivity and proneness to rumination (65–67), and Shipman et al. (68) found poorer emotion regulation skills in neglected children. In the last decade, poor emotion regulation has also been acknowledged as a core feature of eating disorder psychopathology and as a central mechanism leading from pain to impairment (69–72), possibly explaining the association of these criteria with *Maltreatment* in our study. *Sexual Abuse*, but not *Maltreatment*, was associated with dysregulation of bodily sensations, such as pain intensity and hunger/satiation.

### Model comparison

Regarding most criteria, the regression model employing the dimensional approach (model 5) had superior predictive power to the models employing the cumulative (model 1) and selective approach (models 2–4). The fact that model 5 was statistically penalized for having more predictors without containing more information than the other models, corroborates this finding. As to qualitative comparison, the cumulative approach model did not predict alcohol dependency, dysthymia and specific phobia. However, employing the dimensional approach unmasked effects with different signs, which had canceled each other out in model 1. For example, *Household Dysfunction* had a negative effect on the probability of Dysthymia, and *Maltreatment* and *Sexual Abuse* had a positive one, resulting in a non-significant, near-zero effect of the ACE sum score on this criterion. Also, nearly half of the significant coefficients in the selective approach models were unmasked as false positive by the dimensional approach. For example, the significant effects of *Maltreatment* on BMI, abdominal circumference, body fat mass, fat-free body mass, pack years, level of education, Dysthymia, maximum pain intensity, mean pain intensity and cognitive restraint of eating in model 3 where no longer significant after addition of *Household Dysfunction* and *Sexual Abuse* as predictors (model 5). In a similar vein, Schalinski et al. (53) found models containing specific ACE scales such as emotional neglect to be superior to models with indices of global childhood adversity load in the prediction of depressive symptoms in adulthood.

### Strengths and limitations

The design of our study as full survey and the diagnostically diverse sample have likely yielded a representative sample, allowing for generalization of our findings to other clinical populations. Diagnostic sensitivity and specificity should be high as we used specific psychiatric diagnosis established by clinicians using a semi-structured interview. Also, as we penalized the regression models with the ACE dimensions by using Akaike's Information Criterion as comparator although they contained the same information as the ACE sum score, the superior predictive power of the ACE dimensions was likely underestimated in our study.

Limitations to our study are the restriction of the sample population to Western European psychiatric inpatients, the lack of a control group and the medium sample size, especially with regard to the high number of variables. Also, recall bias may have led to overestimation in our clinical sample as adverse childhood experiences were assessed retrospectively (73). For example, retrieval of memories of adverse experiences is likely better in subjects with current depressive episodes due to affect congruence. Then again, forgetting may have led to underestimation of the prevalence of adverse childhood experiences. The negative correlation of age with the ACE sum score seems to point in this direction, although this might also be due to a cohort effect (e.g., divorce was generally less frequent in the 1960s and 70s than in later decades) or a selection effect (high ACE is associated with excess mortality, which lowers the mean ACE of older populations).

Another possible confounding factor of our study is omitted variable bias: Several other studies could improve the ACE questionnaire by adding items focusing on extrafamiliar experiences (e.g., mobbing in school) or witnessing (e.g., of parental violence against siblings) (74–78). Also, we did not statistically control for the effect of other criteria (e.g., the effect of alcohol dependency on the probability of developing a MDD) and did not collect data on the timing, duration or intensity of adverse experiences (9). Lastly, the majority of interval-scaled predictors in our study differed significantly from the normal distribution, violating one of the assumptions of regression.

### Outlook

The dimensional model presented here was built in a bottom-up fashion from rates of co-occurrence of adverse childhood experiences in a diverse clinical sample. Its construct validity needs further vigorous testing, using different samples (e.g., samples from the general population, from other cultures) and different methodologies [e.g., latent class analysis as employed by Brodbeck et al. (43)]. Also, future studies should compare the predictive power and general construct validity of our empirical dimensional model of adverse childhood experiences to other dimensional models, e.g., the theory-derived two-dimensional model by McLaughlin and Sheridan (79) which distinguishes between threat- and deprivation-based childhood experiences.

Regarding the differential impact of the ACE dimensions, future research should try and disentangle the effects of childhood maltreatment and sexual abuse. In our study, the effects specific to the ACE dimension *Sexual Abuse* pertained to regulation of bodily sensations like pain and hunger/satiation, which seems plausible given the more embodied nature of this form of traumatization compared to *Maltreatment*. Following this line of reasoning, sexual abuse might contribute to different psychopathological features of BPS and PTSD than maltreatment. For example, sexual abuse might facilitate feelings of emptiness, dissociation and self-harming behavior *via* poor own body perception whereas maltreatment might contribute to affective instability and difficulty controlling anger *via* deficits in emotion regulation.

## Conclusion

Our data indicate that different dimensions of adverse childhood experiences have qualitatively different consequences for adult mental health and health behavior. Specifically, *Household Dysfunction* was associated with poor health behaviors and poorer socio-economic achievement in adulthood, perhaps mediated by model-based learning of dysfunctional coping strategies. The previously reported associations of maltreatment and sexual abuse with these outcome criteria could not be corroborated and might be due to a type II error due to lack of statistical control for *Household Dysfunction* in those studies. Both *Maltreatment* and *Sexual Abuse* predicted BPD, PTSD and suicidal behavior. However, the two ACE dimensions showed sufficiently divergent validity to warrant separate consideration: *Maltreatment* was associated with affective and anxiety disorders such as social phobia, panic disorder and major depressive disorder, perhaps mediated by poor emotion regulation. In contrast, *Sexual Abuse*, but not *Maltreatment*, was associated with dysregulation of bodily sensations, such as pain intensity and hunger/satiation, possibly due to the more embodied nature of this dimension of childhood adversity. Also, we found both quantitative and qualitative evidence for the superiority of the dimensional approach to exploring the consequences of adverse childhood experiences in comparison to the cumulative and selective approaches. The dimensional approach seems to combine the best of two worlds—the comprehensiveness of the cumulative approach and the distinctiveness of the selective approach.

## Author contributions

WG, KK, and US contributed conception and design of the study. WG, KK, and AS acquired the data and organized the database. AW, AS, and MH performed the statistical analysis. AW wrote the first draft of the manuscript. All authors contributed to manuscript revision, read and approved the submitted version.

### Conflict of interest statement

The authors declare that the research was conducted in the absence of any commercial or financial relationships that could be construed as a potential conflict of interest.
